# Genetic diversity and population structure of feral rapeseed (*Brassica napus* L.) in Japan

**DOI:** 10.1371/journal.pone.0227990

**Published:** 2020-01-16

**Authors:** Ruikun Chen, Ayako Shimono, Mitsuko Aono, Nobuyoshi Nakajima, Ryo Ohsawa, Yosuke Yoshioka

**Affiliations:** 1 Graduate School of Life and Environmental Sciences, University of Tsukuba, Tsukuba, Ibaraki, Japan; 2 Faculty of Science, Toho University, Funabashi, Chiba, Japan; 3 Center for Environmental Biology and Ecosystem Studies, National Institute for Environmental Studies, Tsukuba, Ibaraki, Japan; 4 Faculty of Life and Environmental Sciences, University of Tsukuba, Tsukuba, Ibaraki, Japan; Southwest University, CHINA

## Abstract

Rapeseed (*Brassica napus* L.) is one of the most economically important oilseed crops worldwide. In Japan, it has been cultivated for more than a century and has formed many feral populations. The aim of this study was to elucidate the genetic diversity of feral rapeseeds by genotyping 537 individuals (among which 130 were determined to be genetically modified) sampled from various regions in Japan. Analysis of 30 microsatellite markers amplified 334 alleles and indicated moderate genetic diversity and high inbreeding (expected heterozygosity, 0.50; observed heterozygosity, 0.16; inbreeding coefficient within individuals, 0.68) within the feral populations. The Mantel test showed only an insignificant weak positive correlation between geographic distance and genetic distance. Analysis of molecular variance showed a greater genetic diversity among individuals than between populations. These results are in accordance with population structure assessed by using principal coordinate analysis and the program STRUCTURE, which showed that the 537 individuals could be assigned to 8 genetic clusters with very large genetic differences among individuals within the same geographic population, and that among feral individuals, many are closely related to rapeseed accessions in the NARO Genebank but some have unknown origins. These unique feral rapeseeds are likely to be affected by strong selection pressure. The results for genetically modified individuals also suggest that they have two different sources and have a considerable degree of diversity, which might be explained by hybridization with nearby individuals and separation of hybrid cultivars. The information obtained in this study could help improve the management of feral rapeseed plants in Japan.

## Introduction

Rapeseed (*Brassica napus* L.; genome AACC, 2*n* = 38) is one of the most economically important oilseed crops worldwide with 75 million tonnes produced per year [1,2]. Rapeseed constitutes 13% of global per capita oil consumption for culinary purposes [1,2]. Japan is a rapeseed producing country, with cultivation dating back to the late 19th century [3,4]; the average annual consumption in Japan is about 2.4 million tonnes [2]. After the 1930s government-led promotion work, rapeseed cultivation area and production volume greatly expanded in Japan [3]. This expansion has slowed since the late 1960s due to the permission to import rapeseed, and Japanese rapeseed consumption is now dependent on imports [5]. At present, Japan produces about 3000 t of rapeseed seeds per year, which accounts for only 0.1% of total consumption [1]. Production is concentrated in only a few areas [6]: e.g., Hokkaido, the northernmost island of Japan, where large-scale agricultural production systems are used [1]. Although rapeseed production has almost ceased in Japan, feral rapeseed populations continue to be present in various regions [1–3,7]. Most of these populations are distributed on roadsides or riversides. In both Japan and other rapeseed-producing countries, feral populations are able to complete a whole life cycle [5,8,9]. Many studies have found that the existence of these feral populations is not stable, and that they are more likely to come from spillage during transportation and/or the escape of nearby crops [8–12]; these possibilities have not been confirmed, owing to a lack of knowledge of the genetic background of the feral populations.

Canada, the largest source of imports in Japan (>90% of total imports), has produced genetically modified (GM) rapeseed since the late 1990s; in recent years, the percentage of GM rapeseed in Canada has reached nearly 100% [13,14]. In a survey conducted in 2016–2011, Japanese feral rapeseed was found to include GM individuals [10,15]. Because of the decline in Japanese rapeseed production, it is easy to speculate that the existing feral rapeseed populations in Japan are caused by spillage of imported rapeseed during transportation. However, in the above survey and another survey conducted in 2014, few feral rapeseed populations were found to be GM [15,16]. In response to this mismatch, it has been suggested that the non-GM rapeseed individuals may come from non-GM rapeseed imported many years ago or from sources other than oilseed imports [15]. However, the sources of GM rapeseeds and their relationship with non-GM individuals in Japan are not known.

In a previous study, we performed multiplex PCR using single-locus simple sequence repeat (SSR) primers to analyze the genetic diversity of Japanese and overseas rapeseed accessions preserved in National Agriculture and Food Research Organization (NARO) Genebank [17]. By identifying and then using a number of SSR primers with high reliability and rich polymorphism, we found distinctions between Japanese and overseas rapeseed accessions. Here, we used the same primers to analyze Japanese feral rapeseed populations.

This study had three objectives: (1) Use SSR markers to characterize the genetic diversity of feral rapeseed populations collected from various locations in Japan. (2) Compare the SSR marker-based genotype data of feral rapeseed and NARO Genebank resources to explore the possible sources of feral rapeseed. (3) Ultimately, explore the possible origin of GM feral rapeseed.

## Materials and methods

### Plant material

**Fig 1:**
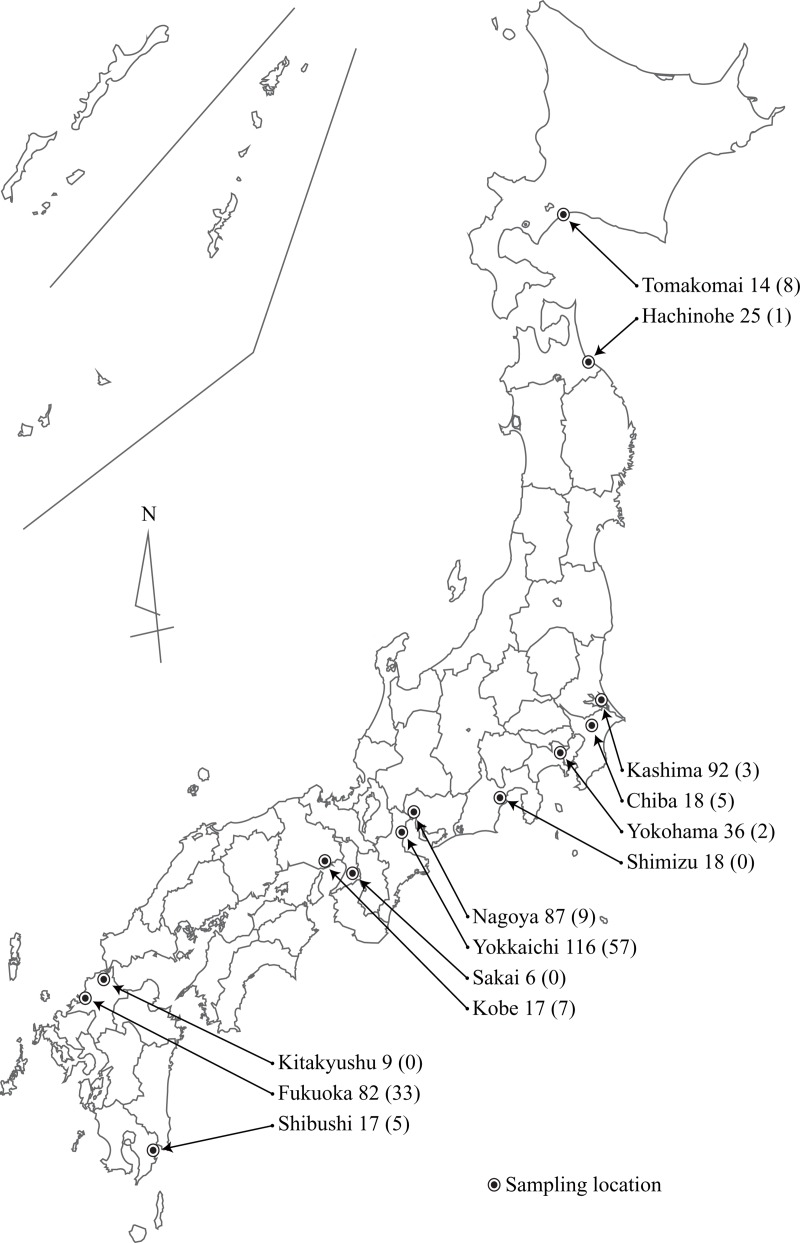
Map of the sampling locations for Japan feral rapeseed. Number of samples, and number of GM (genetically modified) samples in parentheses, of each location follow the location names.

Samples of feral *B*. *napus* (from 537 individuals) were obtained from the National Institute for Environmental Studies (NIES) of Japan (143 accessions) and the Ministry of Agriculture, Forestry and Fisheries (MAFF) of Japan (394 accessions). The seeds of the former materials were collected in 2005 [10], and the leaves of the latter materials were collected in 2015. The species of each collected sample was identified by its morphological characteristics. Samples were tested for the presence of herbicide tolerance proteins (CP4 EPSPS and PAT) by using a kit from Strategic Diagnostics Inc. (Newark, DE, USA), and the presence of herbicide tolerance genes was confirmed by PCR [10]. If at least one of the above proteins were detected, the individual was considered genetically modified. Overall, the individuals were sampled from 13 locations in Japan, covering a diverse geographical range (Fig 1).

### Genotyping

Seeds were germinated on moistened filter paper in 9-cm Petri dishes and then transplanted into 72-cell trays filled with Nippi-Engei-Baido-1gou soil (Nihon Hiryo Co., Ltd., Tokyo, Japan). Seedlings were grown in the farm of Tsukuba Plant Innovation Research Center, University of Tsukuba, until the first true leaves had fully expanded. Genomic DNA was extracted from the first true leaf of each seedling and the collected leaves by using the DNeasy Plant Mini Kit (Qiagen, Hilden, Germany).

Thirty single-locus SSR markers screened in our previous study [17] were used. PCR mixtures (10 μl) contained template DNA (10 ng), 1× KAPA 2G buffer A (KAPA Biosystems Inc., Woburn, MA, USA), 200 nM dNTP, 0.5 mM MgCl_2_, 0.1 U KAPA 2G Fast DNA polymerase, 2 pmol reverse primer, and 0.5 pmol forward primer. The forward primers were 5′-labeled with the fluorescent dyes 6-FAM, VIC, NED, or PET [18]. PCR was performed in a C1000 Thermal Cycler (Bio-Rad Laboratories, Inc., Hercules, CA, USA) as follows: initial denaturation at 94°C for 3 min; 30 cycles of 94°C for 20 s, 54°C for 30 s, and 62°C for 30 s; 3 cycles of 94°C for 20 s, 49°C for 10 s, and 72°C for 5 s; and a final extension at 72°C for 10 min. The size of the amplified fragments was estimated by using an automated DNA analyzer (model 3130xl) with a GeneScan-600LIZ size standard and GeneMapper v. 4.0 software (all from Thermo Fisher Scientific, Inc., Waltham, MA, USA).

### Genetic analysis

The number of alleles, major allele frequency, polymorphism information content (PIC), *F* statistics indices, and Nei’s genetic distance [19] were calculated for all feral rapeseed individuals, and for each geographic population separately, in PowerMarker v. 3.25 software [20]. Observed heterozygosity (*H*_o_) (the number of heterozygotes for one locus divided by the total number of observed individuals), expected heterozygosity (*H*_e_), and Shannon’s information index (*I*) were calculated in GenAlEx v. 6.502 software [21].

Hierarchical analysis of molecular variance (AMOVA) and the Mantel test of the association between genetic distance and geographic distance were calculated in Arlequin v. 3.5.2.2 software [22]. The geographic distance was measured in Google Earth (https://www.google.com/earth/) from the sampling location information.

### Population structure analysis

Genotype data for the SSR markers were analyzed in the model-based STRUCTURE v. 2.3.4 software [23] to determine the most probable number of clusters (*K* value) and to assign rapeseed individuals to different clusters. The *K* value was determined by running an admixture and related frequency model with *K* = 1 to 15 (10 replications per *K* value); the burn-in period of each run and the Monte Carlo Markov Chain lengths were both set to 100,000. The website program STRUCTURE HARVESTER was used to estimate the optimal *K* value [24]; this program follows the Δ*K* method of Evanno et al. [25].

### Comparison with rapeseed accessions in NARO Genebank

To reveal the population structure, we combined the genotype data from the current study with the NARO Genebank data for 582 accessions from our previous study that used the same markers [17]. Then principal coordinates analysis (PCoA) was conducted in GenAlEx v. 6.502 software, and STRUCTURE v. 2.3.4 was used with the settings described in the previous section.

## Results

### Genetic diversity of feral rapeseed

The 30 SSR markers amplified a total of 334 alleles, ranging from 2 (BoEMS0049) to 39 (BrGMS0070) alleles per marker (mean, 11.13; Table 1). The mean genetic diversity indices in the feral populations were 0.52 for major allele frequency, 0.62 for *H*_e_ (indicating moderate to high levels of polymorphism), 0.16 for *H*_o_ (range, 0.05–0.26; indicating that almost all individuals were highly homozygous: i.e., homozygous at all markers), and 0.61 for PIC (indicating that these markers were informative). The F_IS_, which is the deviation of the actual frequency of the genotype from the theoretical expected frequency in the population, was 0.68 (range, 0.52–0.86), indicating a lack of heterozygosity across all markers in the feral populations, and implying that the genotype frequencies at all markers may deviate from Hardy–Weinberg equilibrium in the feral populations.

**Table 1:** Diversity indices of the 30 SSR markers used for genotyping of 537 feral rapeseed accessions.

Marker name	Chromosome	No. of alleles	Major allele frequency	*H*_o_	*H*_e_	PIC	F_IS_
BrGMS4028	A1	8	0.47	0.17	0.65	0.58	0.55
BrGMS4031	A1	16	0.42	0.20	0.76	0.73	0.68
BRAS084	A1	39	0.16	0.20	0.92	0.91	0.60
BrGMS1411	A2	8	0.51	0.22	0.66	0.61	0.67
BrGMS0667	A2	3	0.62	0.14	0.47	0.36	0.73
BrGMS2498	A3	5	0.69	0.12	0.45	0.38	0.84
sN2025	A4	13	0.51	0.18	0.69	0.66	0.66
BrGMS2252	A5	5	0.77	0.05	0.38	0.36	0.65
BrGMS0070	A5	39	0.20	0.23	0.89	0.88	0.78
BnEMS0753	A6	11	0.55	0.21	0.65	0.63	0.73
BrGMS3750	A6	6	0.49	0.20	0.65	0.59	0.80
BrGMS3837	A7	14	0.50	0.10	0.68	0.65	0.61
BnEMS0620	A7	6	0.68	0.12	0.48	0.42	0.75
BrGMS0742	A8	15	0.47	0.17	0.71	0.68	0.56
BnGMS0281	A9	8	0.44	0.21	0.62	0.55	0.69
BrGMS3857	A10	11	0.54	0.16	0.63	0.58	0.57
BrGMS3688	A10	7	0.34	0.25	0.74	0.69	0.83
BrGMS0086	A10	13	0.39	0.24	0.77	0.74	0.62
BnGMS271	C1	8	0.47	0.15	0.64	0.57	0.80
BoGMS2016	C2	14	0.39	0.13	0.77	0.74	0.65
BoEMS0016	C2	7	0.55	0.11	0.57	0.49	0.59
BoGMS0660	C2	6	0.72	0.06	0.43	0.37	0.61
BnGMS0289	C3	14	0.49	0.26	0.71	0.69	0.67
BnGMS347	C4	6	0.39	0.12	0.67	0.61	0.78
BoGMS0037	C5	12	0.78	0.11	0.38	0.36	0.58
BoGMS1909	C6	16	0.36	0.18	0.80	0.77	0.78
BnGMS0353	C6	8	0.75	0.07	0.40	0.36	0.57
BoEMS0049	C7	2	0.64	0.05	0.46	0.35	0.86
BnGMS0336	C8	9	0.45	0.24	0.67	0.61	0.76
BoGMS0525	C9	5	0.85	0.10	0.26	0.25	0.57
Average		11.13	0.52	0.16	0.62	0.57	0.68

*H*_o_, observed heterozygosity; *H*_e_, expected heterozygosity; PIC, polymorphism information content; F_IS_, inbreeding coefficient within individuals. Detailed marker information is available in S1 Table.

The genetic diversity indices for geographic populations are summarized in Table 2. The average number of alleles per marker ranged from 1.80 (Kitakyushu) to 6.67 (Nagoya). The ranges for *H*_e_ and PIC in the 13 populations were 0.19–0.63 and 0.16–0.59, respectively; the Kitakyushu population had the lowest values and the Shimizu population had the highest. The *H*_o_ values ranged from 0.06 (Kitakyushu) to 0.27 (Shibushi). In all populations, *H*_o_ was lower than *H*_e_, and the inbreeding coefficient within individuals F_IS_ in each population was substantially greater than 0 (range, 0.53–0.87), implying that each population may deviate from Hardy–Weinberg equilibrium.

**Table 2:** Genetic diversity estimates in feral rapeseed for each analyzed geographic population.

Population	*N*	*N*_a_	MAR	*H*_o_	*H*_e_	*I*	PIC	F_IS_
Tomakomai	14 (8)	3.50	0.63	0.21	0.47	0.85	0.42	0.57
Hachinohe	25 (1)	3.83	0.62	0.14	0.49	0.89	0.43	0.67
Kashima	92 (3)	5.87	0.61	0.10	0.51	0.97	0.45	0.81
Chiba	18 (5)	4.23	0.61	0.14	0.51	0.97	0.46	0.71
Yokohama	36 (2)	4.87	0.54	0.07	0.57	1.09	0.51	0.87
Shimizu	18	5.40	0.47	0.23	0.63	1.30	0.59	0.67
Nagoya	87 (9)	6.67	0.53	0.18	0.60	1.24	0.56	0.71
Yokkaichi	116 (57)	5.20	0.62	0.18	0.50	0.97	0.45	0.64
Kobe	17 (7)	2.97	0.67	0.15	0.43	0.76	0.38	0.68
Sakai	6	2.50	0.71	0.16	0.39	0.65	0.34	0.53
Kitakyushu	9	1.80	0.85	0.06	0.19	0.33	0.16	0.71
Fukuoka	82 (33)	6.33	0.58	0.17	0.56	1.12	0.51	0.68
Shibushi	17 (5)	4.83	0.50	0.27	0.63	1.23	0.58	0.56
Overall	537	4.46	0.61	0.16	0.50	0.95	0.45	0.68

*N*, number of samples and number of GM samples (in parentheses); *N*_a_, number of alleles per locus; MAR, major allele richness; *H*_o_, observed heterozygosity; *H*_e_, expected heterozygosity; *I*, Shannon’s information index; PIC, polymorphism information content; F_IS_, inbreeding coefficient.

**Fig 2:**
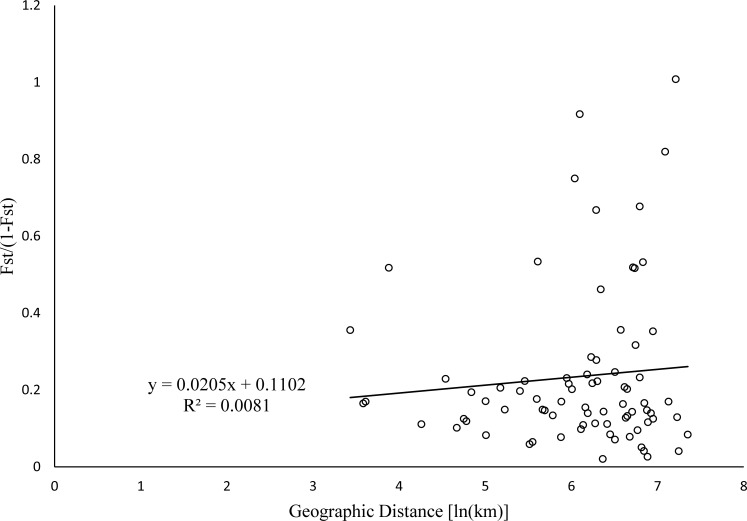
Relationship between geographic distance and genetic distance (F_ST_/(1 − F_ST_)) for Japanese feral rapeseed populations.

The pairwise genetic differentiation coefficient (F_ST_) ranged from 0.038 (Fukuoka and Yokkaichi) to 0.566 (Kitakyushu and Tomakomai) (Table 3). Among the 13 populations, the Kitakyushu population showed a relatively high differentiation from the other populations (all pairwise F_ST_ > 0.4). A similar tendency was seen for the Nei’s genetic distance: the Kitakyushu population showed a relatively high distance from the other populations (mean genetic distance, 0.38; Table 3). The Mantel test showed only an insignificant weak positive correlation (*r* = 0.09, *p* = 0.32) between geographic distance and genetic distance (Fig 2).

**Table 3:** Estimates of pairwise F_ST_ (below diagonal) and Nei’s genetic distance (above diagonal) among feral rapeseed geographic populations.

	Tomakomai	Hachinohe	Kashima	Chiba	Yokohama	Shimizu	Nagoya	Yokkaichi	Kobe	Sakai	Kitakyushu	Fukuoka	Shibushi
Tomakomai		**0.182**	**0.172**	**0.116**	**0.125**	**0.189**	**0.142**	0.025	**0.111**	**0.261**	**0.502**	**0.039**	**0.077**
Hachinohe	0.223		**0.178**	**0.217**	**0.126**	**0.198**	**0.140**	**0.168**	**0.241**	**0.341**	**0.450**	**0.145**	**0.114**
Kashima	0.136	0.196		**0.141**	**0.092**	**0.164**	**0.145**	**0.178**	**0.222**	**0.194**	**0.347**	**0.128**	**0.122**
Chiba	0.125	0.264	0.120		**0.100**	**0.129**	**0.118**	**0.072**	**0.134**	**0.089**	**0.404**	**0.039**	**0.104**
Yokohama	0.182	0.208	0.134	0.150		**0.111**	**0.061**	**0.129**	**0.168**	**0.188**	**0.341**	**0.087**	**0.048**
Shimizu	0.332	0.358	0.263	0.246	0.233		**0.076**	**0.170**	**0.128**	**0.150**	**0.262**	**0.113**	**0.073**
Nagoya	0.203	0.246	0.171	0.174	0.164	0.179		**0.145**	**0.146**	**0.162**	**0.316**	**0.100**	**0.066**
Yokkaichi	0.073	0.245	0.128	0.068	0.147	0.240	0.166		**0.106**	**0.186**	**0.400**	**0.020**	**0.078**
Kobe	0.249	0.375	0.312	0.275	0.304	0.310	0.289	0.227		**0.262**	**0.428**	**0.098**	**0.102**
Sakai	0.233	0.356	0.179	0.127	0.235	0.317	0.271	0.186	0.379		**0.478**	**0.122**	**0.182**
Kitakyushu	0.566	0.495	0.469	0.451	0.441	0.412	0.448	0.474	0.525	0.437		**0.341**	**0.348**
Fukuoka	0.106	0.248	0.122	0.076	0.138	0.206	0.134	0.038	0.236	0.176	0.452		**0.055**
Shibushi	0.184	0.204	0.176	0.188	0.159	0.258	0.171	0.143	0.297	0.309	0.485	0.132	

Bold type indicates a significant value (*p* < 0.05) calculated over 1000 permutations.

AMOVA of the geographic populations (Table 4) showed that the genetic variation within individuals (27.2%) was larger than that among geographic populations (12.62%). However, the genetic variation among individuals within a geographic population was the highest (60.18%). All of these variance components were highly significant, indicating that genetic differentiation within and among individuals and among populations was significant.

**Table 4:** Hierarchical analysis of molecular variance (AMOVA) for feral rapeseed geographic populations.

Source of variation	d.f.	Percentage of	
		variation (%)	
Among geographic populations	12	12.62	***
Among individuals	524	60.18	***
Within individuals	537	27.20	***

***Significant at the 0.1% level for 1000 permutations.

### Population structure of rapeseed accessions

**Fig 3:**
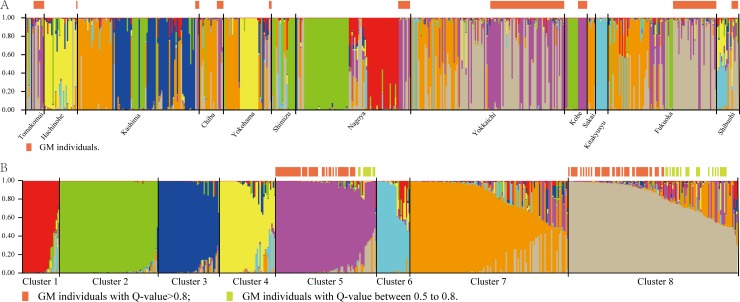
Population structure of 537 feral rapeseed individuals based on 30 SSR markers. At *K* = 8, the 537 individuals were classified into eight groups by STRUCTURE analysis. (A) Ordered by geographic population. (B) Ordered by admixture proportion. GM, genetically modified.

To obtain information about the population structure of feral rapeseed accessions from allelic frequencies, we used two methods: STRUCTURE and PCoA. In the STRUCTURE analysis, the computation of Evanno’s Δ*K* indicated *K* = 8 as the most likely model (S1 Fig), suggesting the presence of 8 main groups: i.e., clusters 1 to 8 (Fig 3). With 0.8 as the likelihood (Q-value) to cluster each accession in the eight clusters, a total of 375 individuals (69.8%) were grouped into one of the eight groups. S2 Table shows the percentage of individuals in each group calculated by Q-value. The Q-value of each individual and population is listed in S3 and S4 Tables, respectively. The Sakai and Kitakyushu populations showed a high proportion (Q-value > 0.8) of membership in a single group (cluster 6; S4 Table), while the individuals of other populations were assigned to different groups or had a high percentage of admixture. The Shibushi population had the highest percentage of admixture (94.1%). The grouping determined by STRUCTURE was not related to the geographic distance between populations. The Mantel test showed no significant isolation by distance, suggesting that geographic distance is not a major factor in the observed population structure, and was consistent with the results of STRUCTURE.

In the combined dataset, all GM individuals were classified into Clusters 5 and 8 (Fig 3B). These two clusters were composed mainly of GM individuals: GM/Total = 58/75 (Cluster 5) and 72/121 (Cluster 8).

**Fig 4:**
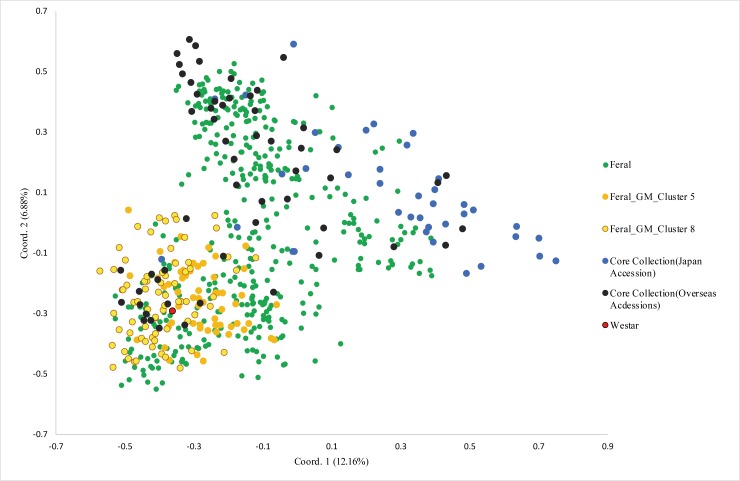
Scatter diagram of PCoA of the NARO Genebank and feral rapeseed samples. Among the NARO Genebank accessions, only the ones belonging to the core collection in NARO Genebank are shown in this figure. Coord., coordinate.

The result of PCoA for the feral rapeseeds and the NARO Genebank accessions included in the core collection constructed in a previous study is shown in Fig 4 [17]. The first two axes of the PCoA explained ~19.04% of the variation in the genetic distance matrix. A bi-dimensional PCoA scatter plot indicated differentiation between the 13 feral rapeseed populations (Fig 4). In the plot, the majority of the feral rapeseed accessions were distributed in areas that had a higher density of overseas accessions than other areas. GM feral rapeseed accessions were also distributed in areas that had a relatively high density of overseas accessions, and maintained considerable diversity. However, geographic differentiation was not observed in the GM feral rapeseeds (S4 Fig).

## Discussion

In this study we explored the genetic diversity and population structure of feral rapeseed populations in Japan by using the SSR markers which we used in our previous study [17]. By genotyping the feral accessions with these SSR markers, we revealed that the feral rapeseed populations have high genetic diversity (Table 1). The high values for number of alleles (334 alleles detected by 30 markers), *H*_e_ (mean, 0.62), and PIC (mean, 0.57) indicate that the SSR markers are suitable for diversity analysis of feral rapeseed. Feral rapeseed populations are found in countries other than Japan [26], but there has been little research on their genetic diversity. We observed that the mean *H*_e_ of the feral populations in Japan is higher than that of populations in Austria (0.62 versus 0.32) [27]. Japan may have feral populations with higher genetic diversity owing to the introduction of different rapeseed cultivars from multiple areas of the world.

The inbreeding coefficient within individuals (F_IS_), which ranges from −1 to +1, indicates the degree of inbreeding. Extremely positive significant values mean that outcrossing predominates in the population, whereas extremely negative significant values mean that inbreeding predominates [28]. The mean F_IS_ value of the feral populations was 0.68 (Table 2), indicating that these populations reproduce mainly by selfing. This notion is supported by the low *H*_o_ of 0.16 and is consistent with the suggestion that although rapeseed is often cross-pollinated, it reproduces mainly by self-crossing [15].

The population genetic differentiation coefficient (F_ST_), which ranges from 0 to 1, is an important indicator of genetic differentiation between populations; values over 0.15 are considered to indicate substantial genetic differentiation [29]. Here, in a pairwise comparison, the differentiation between almost all feral populations was found to be significant (*P* < 0.05; Table 3). Most of the populations, except Kitakyushu, had pairwise F_ST_ values around 0.2, indicating that the differentiation between them and other populations was moderate; the Kitakyushu population had pairwise F_ST_ values of >0.4, indicating strong differentiation. These results show that individuals within these populations were different from those in other populations, indicating that all of the feral populations except Kitakyushu may have originated from several founding events including but not limited to escapes of cultivars with different genetic backgrounds, or from seeds dropped during transportation. Such origin of these populations can also explain why the Mantel test revealed no significant correlation between genetic distance and geographic distance among the different populations. The result of AMOVA hierarchical analysis showed genetic variation mainly among individuals and not between populations, providing further evidence that most feral populations were composed of individuals that differed from each other. The Kitakyushu population showed strong identity in the PCoA and STRUCTURE analysis, and a different genetic background from other populations, perhaps because it had a single source or because only nine samples were collected from it. In a future study, more samples should be collected from the Kitakyushu population to confirm the results.

Both PCoA and STRUCTURE analysis based on the combined dataset of rapeseed accessions in the NARO Genebank and feral rapeseeds (Fig 4; S3 Fig) indicated that many feral individuals had a close relationship to NARO Genebank accessions. However, in PCoA plot, some feral individuals distributed in the regions where are no varieties and landraces, suggesting that they are genetically distant from accessions in the NARO Genebank. Such genetic distence may arise if the germplasm is not included in the Genebank, or as a result of hybridization between different accessions or allele segregation by selfing. Previous small-scale studies of feral rapeseed in England, Germany, and Austria also found that some of the feral individuals were different from the cultivars grown in the same region [9,27,30]; in these studies, the number of cultivars compared was 13, 5, and 19, respectively, whereas the number in the current study was 582. Because landraces are a possible source of feral rapeseed, expanding the range, especially to include those with a long history, may allow determination of the origin of feral rapeseed. However, although there are many early cultivars among the 582 accessions in the NARO Genebank, we found many feral rapeseed individuals different from them. This result shows that these unique feral rapeseeds may be affected by strong selection pressure, or may have originated from other cultivars that were not included in the analysis.

Since the first approved imports of GM rapeseed into Japan, reports have shown that GM rapeseed can cross with wild relatives, thereby spreading the introduced genes [31,32]. Previous surveys of feral rapeseed in Japan showed that the imported GM seedlings could grow to sexual maturity in wild conditions. Here we further analyzed the genetic relationship between GM and non-GM individuals. In the PCoA plot (Fig 4), the GM individuals tended to concentrate together, although the distribution area was still wide; this area centered on a widely used parent of GM rapeseed, ‘Westar’ [33], but no GM feral rapeseed individual was at the same point as ‘Westar’. With the promotion of hybrid rapeseed cultivars in oilseed-exporting countries [34–36], it can be speculated that the GM rapeseed imported into Japan is largely offspring of these cultivars and that the diversity of feral GM rapeseeds in Japan may be derived from the genetic segregation of these hybrids. The importation of many genetically different rapeseed cultivars will directly lead to such a situation. In addition, some studies have detected the transfer of transgenes from GM rapeseed to related *Brassica* species [37,38]. Therefore, GM rapeseed growing in Japan may have hybridized with nearby non-GM rapeseeds (and of course other GM cultivars), resulting in a broad diversity of GM feral populations. In the STRUCTURE analysis of feral rapeseed, GM individuals were placed into two of the eight clusters (Clusters 5 and 8; Fig 3), suggesting that there may be two different sources of GM rapeseed, namely from two different GM cultivars imported into Japan. In the STRUCTURE analysis of the combined dataset of feral rapeseed and Japanese and overseas accessions (S3 Fig), GM individuals were classified into clusters that were composed mainly of feral rapeseeds, and these clusters also contained a considerable number of overseas accessions. This implies that the genetic background of GM feral rapeseed is closer to that of overseas accessions than to that of Japanese accessions.

Surveys of GM crops entering the natural environment for different reasons have been performed in various countries and for multiple crops [30,39]. However, the study of genetic diversity among these transgenic plants living in the wild has not received sufficient attention. Our findings regarding the diversity of GM feral rapeseed in Japan provide a decision-making basis for future GM rapeseed control. It is noteworthy that the genetic diversity of the feral rapeseed population in Japan may change as new rapeseed cultivars (especially GM) are imported from rapeseed-producing countries. The possibility of these changes must be considered in future studies. The next-generation sequencing methods, which provide more information and wider genome coverage than genotyping with SSR markers, will be more helpful for the exploration of specific genetic relationships between individuals.

From the high diversity between and within feral rapeseed populations, we conclude that the source of these populations in Japan is the escape of various cultivars that were grown or imported as oilseed. Furthermore, we demonstrate that some of the feral rapeseed individuals are genetically distant from accessions in NARO Genebank, and that feral GM rapeseed in Japan has a considerable degree of diversity.

## Supporting information

S1 Fig Delta *K* values for different numbers of clusters assumed (*K*) in the STRUCTURE analysis of feral rapeseed individuals. (EPS)

S2 Fig Delta *K* values for different numbers of clusters assumed (K) in the STRUCTURE analysis of feral and Genebank rapeseed individuals. (EPS)

S3 Fig Population structure of 1119 feral and Genebank rapeseed individuals based on 30 SSR markers. At *K* = 4, all individuals were classified into four groups.
(EPS)

S4 Fig Scatter diagram of PCoA of the GM feral rapeseed samples. This figure is an enlargement of part of Fig 4. Samples that belong to different geographical populations are marked with different colors, and 2 clusters inferred by STRUCTURE analysis (See Fig 3) are marked with different shapes of the symbols.
(EPS)

S1 Table Primer information of the 30 SSR markers used in this study. (XLSX)

S2 Table Number and percentage of feral rapeseed individuals in each inferred cluster among the 13 populations. (XLSX)

S3 Table Q-values of each feral individual for 8 inferred clusters. (XLSX)

S4 Table Average Q-values of each population for 8 inferred clusters. (XLSX)
